# Effects of cyclic parenteral nutrition on parenteral-associated liver dysfunction parameters

**DOI:** 10.1186/s12937-017-0289-7

**Published:** 2017-10-04

**Authors:** Jose J. Arenas Villafranca, Miriam Nieto Guindo, Elena Álvaro Sanz, Manuela Moreno Santamaria, Marga Garrido Siles, Jimena Abilés

**Affiliations:** 1 Pharmacy and Nutrition Service, Costa del Sol Hospital, A7, km. 187, 29603 Marbella (Málaga), Spain; 2 C/ Fernando Villalón Edf. Lorcrisur, Bloque n°8, Bajo A, 29670 Marbella (Málaga), Spain

**Keywords:** Liver dysfunction, Parenteral nutrition, Cyclic parenteral nutrition, Liver parameters

## Abstract

**Introduction:**

One of the most common complications of parenteral nutrition (PN) is liver dysfunction (LD). Therapeutic approaches for LD include, among others, administering cyclic parenteral nutrition (cPN), allowing some hours for metabolic rest. The purpose of this study was to evaluate the effectiveness of cPN in treating PN-associated LD.

**Materials and methods:**

A retrospective observational study was carried out at the Costa del Sol Hospital in Spain between 2013 and 2014. The study involved inpatients ≥18 years old prescribed with cPN due to the development of PN-associated LD. The hepatic biochemical parameters measured at baseline and after completion of cPN included aspartate aminotransferase (AST), alanine aminotransferase (ALT), gamma-glutamyltransferase (GGT), alkaline phosphatase (ALP) and total bilirubin (TB). Quantitative values (age, biochemical parameters) were compared using matched Student’s t-test; the mean change in qualitative variables (sex, indication of PN, hepatic comorbidities, presence of insulin in cPN, infection during cPN, management of LD prior to cPN administrarion) was estimated using Mann-Whitney U test, and bivariate correlation between quantitative variables was determined by Spearman’s coefficient of correlation.

**Results:**

Thirty-seven patients met inclusion criteria. All hepatic function parameters except ALP improved after the administration of cPN, with statistically significant differences (*p < 0.05*) in AST GGT and TB.

**Conclusion:**

cPN improves PN-associated LD by restoring abnormal AST, GGT, and BT levels to normal, and reducing ALT levels close to normal. The results obtained suggest that the administration of cPN is effective in reverting PN-associated LD.

## Introduction

Parenteral nutrition (PN) is defined as the intravenous provision of nutrients to patients in whom enteral feeding is insufficient or contraindicated. It may be provided via a central or peripheral vein and may be used as stand-alone nutrition support or as an adjunct to enteral nutrition [1].

Although PN is an effective method of nutrition support, it has been associated with a range of mechanical, septic, and metabolic complications [2]. One of the most common complications of parenteral nutrition is liver disfunction (LD), which is associated with a higher risk of mortality [3]; the reason is that if LD is not successfully treated, it can progress to fibrosis and/or liver cirrhosis [4, 5].

Although changes in hepatic function usually occur during long-term PN (in 20 to 80% of cases), patients who receive PN for a short time frequently develop cholestasis [6]. During the first weeks on PN, the activity of hepatic enzymes increases starting with an elevation in alkaline phosphatase (ALP) and gamma glutamyl aminotransferase (GGT) levels in adults. ALP and GGT levels generally increase during the second week on PN, whereas the rise in transaminase levels occurs later. However, the most widely accepted prognostic factor for LD is unconjugated bilirubin [4].

While the etiology of liver dysfunction (LD) is not fully understood, multiple factors are known to be involved in the development of this disorder. Such factors can be patient-related (liver disease, pancreatitis, obesity, alcoholism, digestive rest, bacterial overgrowth); or nutrition-related, (high carbohydrate intake, continuous supply of nutrients). What is certain is that LD must be treated as soon as possible. Therapeutic strategies for LD include avoiding overfeeding, providing a balanced supply of nutrients, supplying early enteral nutrition, and administering discontinuous parenteral nutrition while allowing some hours for metabolic rest (cyclic parenteral nutrition, cPN) [4].

However, only a few studies have been performed to assess the effectiveness of these strategies. The purpose of this study was to evaluate the effects of cPN on liver function parameters.

## Materials and methods

A retrospective observational 15-month study was carried out between 2013 and 2014 in the 350-bed Costa del Sol Hospital, Spain, which attends a population of 404,426 inhabitants. The study involved inpatients ≥18 years who initially received continuous parenteral nutrition and were subsequently prescribed with cPN following a decision of the team of artificial nutrition based on the local PN management protocol. Exclusion criteria included: severe liver disease evidenced by abnormal biochemical hepatic parameters, a history of renal failure, death during treatment or a duration of cPN ≤ 4 days. Data were collected from clinical records.

According to the local protocol, nutrition support consists of a lipid emulsion comprising soy oil, MCT, olive oil and fish oil (SMOF). Nutrition supply was individualized following the guidelines of recognized scientific societies [1, 7]. Glucose and lipid intake never exceeded 5 and 1.5 g/kg/d, respectively. Initially, all patients received continuous PN and, following our local protocol, a series of interventions were performed prior to the administration of cPN, notably: taurine was added to the amino acid solution; the dietary carbohydrate/lipid ratio in PN composition was reverted (in general terms, the standard carbohydrate/lipid ratio is 60:40 or 70:30; however, in a context of LD, it is recommended that the total amount of dextrose is reduced, thereby reverting the standard ratio), and the total carbohydrate and/or lipid supply was reduced.

cPN was only prescribed when LD parameters became abnormal. Specifically and, in accordance with previous studies, liver dysfunction (LD) was defined as: a) Cholestasis: alkaline phosphatase (ALP) > 280UI/L, gamma-glutamyltransferase (GGT) > 50UI/L ó total bilirubin (TB) > 1.2 mg/dL; b) Hepatic necrosis: Aspartate aminotransferase (AST) > 40UI/L, alanine aminotransferase (ALT) > 42UI/L and c) Mixed pattern: ALP > 280UI/L, GGT > 50UI/L or TB > 1.2 mg/dL plus AST > 40 IU/L or ALT > 42UI/L [8].

Study variables included: a) Qualitative parameters: sex, diagnosis at admission, indication of PN, hepatic comorbidities, start date of cPN, presence of insulin in cPN, infection during cPN and management of liver dysfunction prior to the administration of cPN (addition of taurine to the amino acid solution, reversal of the dietary carbohydrate/lipid ratio of PN composition, and reduction of carbohydrate and/or lipid supply). b) Quantitatives: age and biochemical parameters including AST, ALT, GGT, ALP, and TB that were measured at baseline and at completion of cNP using validated techniques. These parameters were monitored from the initiation of cPN until the end of the cycle, when biomarkers became stable. To estimate the reduction in TB levels, only the TB values ≥1.2 mg/dL at the start of cPN were considered.

Descriptive analysis was performed using central tendency, dispersion, and distribution for quantitative variables, and frequency distribution for qualitative variables. Baseline and post-cPN hepatic values were compared using matched Student’s t-test; the mean change in qualitative variables was estimated using Mann-Whitney U test. Bivariate correlation between quantitative variables was determined by Spearman’s coefficient of correlation. When paired samples exceeded 30 individuals, parametric statistical tests were performed. A *p* value <0.05 was considered statistically significant. Data collection and analyses were performed using SPSS v15.

## Results

Of the 557 patients who received PN during the study period, 56 were prescribed cPN (10%), of whom 37 met the inclusion criteria. Demographic data are shown in Table 1. All patients were considered by Costa del Sol Hospital nutritionists to have a good nutritional status. The mean duration of continuous parenteral nutrition until prescription of cPN was 15.2 ± 11.2 days. Most patients received cPN for 12 h prior to the infusion of saline solution within the following 12 h until enteral nutrition was initiated. The mean duration of cPN was 12.8 ± 12.7 days (range 3 to 42 days).

**Table 1:** General characteristics of the study population (*n* = 37)

Age (years)	61.2 ± 13.7
Gender (Man/Woman)	40.5%/59.5%
Weight (Kg)	65.6 ± 19.4
BMI (kg/m^2^)	24.0 ± 6.5
Indications for PN	
Major digestive surgery	29.7%
Bowel obstruction	21.6%
Severe Acute Pancreatitis	18.9%
Gastrointestinal Occlusion	10.8%
Need for digestive rest	8.1%
High output fistula	8.1%
Uncontrollable vomiting	2.7%
Comorbidities (No. of patients)	
Liver angioma	1
Cholecystectomy	3
Cholelithiasis	3
Dyslipidemia	4
Alcoholism	3
HBV	1
None	24 (65%)

*HBV* Hepatitis B Virus, *PN* Parenteral nutrition, *IBD* Intestinal bowel Disease

In all, 43.2% of patients received some treatment to manage LD prior to cPN. The lipid and carbohydrate supply was reduced in 8% of patients, and taurine was added to the amino acid emulsion in 38%. However, no significant improvements were achieved in any hepatic parameter through these interventions.

Following cPN, an improvement in all hepatic function parameters except ALP was observed, as shown in Table 2, with statistically significant differences in AST (*p < 0.05*), GGT (*p < 0.05*) and TB (*p < 0.05*). No correlation was observed between improvements in hepatic function parameters and any demographic variable (age, sex, weight and BMI), neither with cPN duration.

**Table 2:** Hepatic parameter values before and after the administration of cyclic parenteral nutrition (*n* = 37)

Hepatic parameter values at the start of cPN	Pre-cPN value	Post-cPN value	*p*
AST (UI/L) (mean ± SD)	78.2 ± 89.1	34.8 ± 17.3	0.004
ALT (UI/L) (mean ± SD)	132.5 ± 245.4	55.0 ± 36.2	0.058
GGT (UI/L) (mean ± SD)	539.5 ± 372.9	414.2 ± 321.5	0.040
Alkaline phosphatase (UI/L) (mean ± SD)	238.3 ± 156.8	239.6 ± 199.9	0.968
Total Bilirubin >1.2mg/dL (mg/dl) (*n* = 5) (Median (IQR))	1.5 (1.2–6.2)	0.8 (0.2–3.1)	0.047

*cPN* cyclic parenteral nutrition, *IQR* InterQuartile rank

In total, 35% of patients had a hepatic comorbidity (Table 1). Improvements in hepatic parameters were not found to be correlated with any previous comorbidity. The only exception was TB, which reduction was significantly greater in patients with high baseline TB levels (Table 1). No correlation was observed either between the development of infection (54%), insulin supply through cPN (43%), enteral stimulus (54%) and the reduction in hepatic parameter values.

## Discussion

Some studies have been performed to assess the effectiveness of administering cPN to manage hepatic complications but changes in hepatic parameter values have never been explore during cPN, and our study, also reveals that the administration of cPN (12 days on average) objectively reduced significantly abnormal AST and TB values to normal levels and ALT close to normal levels. However, no changes were observed in ALP. The reduction of hepatic parameters during cPN has a clear clinical benefit in patients, as elevated transaminase levels caused by steatosis can progress to fibrosis or hepatic cirrhosis in the long term.

The administration of cPN at night was proposed by Scribner et al. in 1970 [9]. A randomized study in adults [10] demonstrated that cPN mimicked better the physiologic function of nutrient metabolism while the lipogenesis caused by fasting was prevented, thus improving the clearance of the fatty acids accumulated during continuous PN. Another retrospective study in neonates assessing the prophylactic administration of cPN versus standard PN demonstrated that cPN reduced the incidence or delayed the development of hepatic abnormalities [11]. Moreover, a revision of the metabolic effects of cPN in adults and children concludes that cPN infusion has a favorable risk–benefit profile in most patients receiving long-term PN [12].

There is controversy concerning the etiology and pathogenesis of LD [2]. Continuous PN may be a predisposing factor for excess insulin levels, which cause the accumulation of fat in the liver [2]. However, the causes of hepatic and biliary abnormalities induced by continuous PN have not been identified yet [3]. Hepatic parameters should be continuously monitored in patients receiving PN to prematurely detect and treat any potential liver dysfunction. For this reason, our protocol includes the weekly monitoring of hepatic parameters. It is widely known that the most sensitive markers of cholestasis are GGT and unconjugated bilirubin, although none of these parameters is specific [4]; consequently, we follow Grau et al. LD classification instead of just waiting for an abnormal elevation of GGT and BT values.

Preventive measures are routinely implemented in our hospital to protect the liver. Thus, patients receive individualized PN comprising 20% lipid emulsions, with supplies not exceeding the intake recommended in the local protocol [13]. All in all, 10% of our patients developed liver dysfunction during the study period. Even so, the incidence of LD in our hospital is significant below the rate (78%) reported by Pallarés et al. [14] or the rate reported by Aaron R. Jensen (35%) in children after 12–18 days on PN [11]. Such difference might be explained by the fact that the PN initially administered in our hospital includes measures for the prevention of liver dysfunction, added to the fact that patients with a previous liver dysfunction were excluded from the study (15 patients). According to our protocol, these therapeutic measures must be adopted as soon as hepatic abnormalities are detected, which contradicts a recent study that suggests a benefit from early initiation of cPN [15]. Such measures include avoiding overfeeding, the early administration of enteral nutrition, the administration of cPN (8–12 h) [3], and the addition of taurine in PN. Taurine supplementation has been proven to improve biliary flow by increasing the rate of biliary acid secretion [16–18] which accumulates as a result of continuous PN [19].

Although a series of interventions were performed in our study prior to the administration of cPN following our local protocol, none of these strategies was associated with a significant reduction in hepatic function parameter values prior to the administration of cPN. Therefore, cPN was the most effective strategy for the normalization of biochemical hepatic parameters.

The identification of patients with predisposing factors for liver dysfunction is essential [13]. However, in this study, no correlation was observed between previous hepatic comorbidities and biochemical changes following cPN, except for TB. This might be explained by the fact that increases in TB generally occur later than other hepatic parameters [20], and the patients who had no comorbidities were administered early cPN, which prevented an increase in TB levels.

There are other factors that predispose patients to develop hepatic complications, especially in the case of sepsis or malnutrition [8, 21]. Over 50% of our patients developed an infection confirmed on culture. However, the good nutritional status of our patients (as determined by nutritionists) eliminates the possibility that malnutrition was a predisposing factor for LD.

A limitation of this study is the small sample size, which makes it difficult to perform an analysis with enough statistical power. Nevertheless, this study represents a potential for further research in this area.

## Conclusion

The administration of cPN was effective in reducing significantly abnormal hepatic biochemical values to normal levels, including AST, GGT, and TB, and in reducing almost significantly ALT levels. However, no changes were observed in ASP. The results obtained suggest that the administration of cPN is effective in reverting parental-associated liver dysfunction.

## Abbreviations

ALP: Alkaline phosphatase
ALT: Alanine transaminase
AST: Aspartate aminotransferase
BMI: Body mass index
cPN: cyclic Parenteral Nutrition
GGT: Gamma-glutamyltransferase
LD: Liver disfunction
PN: Parenteral nutrition
TB: Total bilirubin
